# Genome Sequence of Hydrothermal Arsenic-Respiring Bacterium *Marinobacter santoriniensis* NKSG1^T^

**DOI:** 10.1128/genomeA.00231-13

**Published:** 2013-05-09

**Authors:** Kim M. Handley, Mathew Upton, Scott A. Beatson, Marina Héry, Jonathan R. Lloyd

**Affiliations:** Williamson Research Centre for Molecular Environmental Science and the School of Earth, Atmospheric and Environmental Sciences, the University of Manchester, Manchester, United Kingdoma;; Microbiology and Virology Unit, Institute of Inflammation and Repair, the University of Manchester, Manchester, United Kingdomb;; School of Chemistry and Molecular Biosciences, the University of Queensland, Brisbane, Queensland, Australiac

## Abstract

*Marinobacter santoriniensis* NKSG1^T^ originates from metalliferous marine sediment. It can respire and redox cycle arsenic species and perform mixotrophic, nitrate-dependent Fe(II) oxidation. The genome sequence, reported here, will help further elucidate the genetic mechanisms underlying these and other potential biogeochemically relevant functions, such as arsenic and mercury resistance and hydrocarbon degradation.

## GENOME ANNOUNCEMENT

*Marinobacter santoriniensis* NKSG1^T^ was isolated from temperate shallow marine hydrothermal sediment at Santorini, Greece (1). The bacterium is facultatively anaerobic and thermotolerant. It respires nitrate while using organic acids (e.g., acetate, lactate, and pyruvate), grows aerobically on simple and complex organic substrates, uses fumarate as electron donor or acceptor, and ferments lactate (1). Currently, strain NKSG1^T^ is the only *Marinobacter* isolate known to metabolize arsenic species (aerobically-anaerobically) (2), and it is among two with a demonstrated ability to oxidize iron (1, 3). Its metabolic abilities suggest it is well adapted to exploit the iron- and arsenic-rich environment from which it was cultivated. However, the bacterium lacks the respiratory arsenate reductase, Arr, used by other bacteria (2, 4). *Marinobacter* comprises a genus of metabolically flexible and ubiquitous marine *Gammaproteobacteria* that colonize diverse habits ranging from polar ice to deep-sea hydrothermal sediments and saline terrestrial environments (5). The genus includes species that degrade hydrocarbons and denitrify (6) and respire or enzymatically transform metal(loid)s, such as arsenic, iron, and manganese (1–3, 7); however, the distribution of these functions across the genus is unclear. Analyses of genome sequences may improve our understanding of this genus and its role in biogeochemical cycling in the marine environment. Currently there are 9 other publically available genomes for characterized *Marinobacter* species and a further 5 for uncharacterized members (3, 7–9).

Genome sequencing was performed using the Roche 454 GS-FLX sequencer. Sequencing yielded 114,423,329 bp of DNA, 154,989 shotgun reads ~397 bp long, 394,551 mate-pair reads with an average insert length of 3.9 kb, and ~28 times genome coverage. Assembly was performed using GS *de novo* assembler version 2.0.00.20 (Roche). The draft assembly comprises a total of 38 contigs >200 bp long, with an N_50_ of 293,792 bp. The chromosome comprises a single 4,063,878 bp scaffold consisting of 28 contigs, with 10 unscaffolded contigs totaling 14,508 bp in length. Open reading frames were identified in all scaffolded contigs, and 3,693 predicted proteins were annotated using NCBI’s Prokaryotic Genomes Automatic Annotation Pipeline (PGAAP). The calculated GC content of the draft genome is 58.3%, comparable to the high-pressure liquid chromatography (HPLC)-determined value of 58.1% (1).

In culture, strain NKSG1^T^ accumulates nitrite with nitrate amendment (1). Comparably, the genome contains genes indicative of dissimilatory nitrate reduction (*narIJHGK*) and denitrification from nitric oxide (*norBA* and *nosLYFDZR*), but only assimilatory nitrite reduction (*nirDB*). It possesses arsenite oxidase *aioAB* (10) and expresses *aioB*/*aoxB* during mixotrophic arsenite oxidation (2). We identified no respiratory arsenate reductase, although the strain performs dissimilatory arsenate reduction (2). The genome includes *Escherichia coli*-like *arsC* (closely related to several other *Marinobacter* species), and the related genes *yffB*, *arsH*, and *arc3*, for nonrespiratory arsenate reduction and efflux (11,12). It further contains genes suggestive of mercury resistance (*merRTA*) (13), propionate fermentation via the methylmalonyl-coenzyme A (CoA) pathway (14), and hydrocarbon and solvent degradation (e.g., alkane 1-monooxygenase, cyclohexanone monooxygenase, and 2-nitropropane dioxygenase) (15–17).

### Nucleotide sequence accession numbers.

The genome sequence has been deposited at DDBJ/EMBL/GenBank under the accession number APAT00000000. The version described in this paper is the first version, accession number APAT01000000.
